# Clinical profile and outcome of bipolar disorder patients receiving electroconvulsive therapy

**DOI:** 10.1192/j.eurpsy.2021.527

**Published:** 2021-08-13

**Authors:** W. Bouali, R. Omezzine Gniwa, R. Ben Soussia, A. Hadj Mohamed, L. Zarrouk

**Affiliations:** Department Of Psychiatry, University Hospital Of Mahdia, Tunisia., Psychiatry, Mahdia, Tunisia

**Keywords:** Bipolar disorder, electroconvulsive therapy, mania, depression

## Abstract

**Introduction:**

Bipolar disorder (BD) is a serious and extremely recurrent illness frequently associated with cognitive and functional deterioration that poses many treatment challenges. However, over the years, with the evolution of more and more mood stabilizers and neuroleptics, there were controversies surrounding the use of electroconvulsive therapy (ECT).

**Objectives:**

The present study was an attempt at studying the clinical profile of BD patients who receive ECT and to study its effectiveness.

**Methods:**

Retrospective data were collected from all bipolar patients submitted to acute ECT treatment, between June 2015 and June 2016, at the Department of Psychiatry of the University Hospital of Mahdia, Tunisia.

**Results:**

During the study period, among all the patients who received ECT, 47% were diagnosed to have bipolar disorder. ECT was administered most commonly for mania with psychotic symptoms, followed by severe depression with psychotic symptoms. Most of patients showed more than 65% response (based on reduction in the standardized rating scales) with ECT. Few patients (18.7%) reported some kind of side effects.

**Conclusions:**

ECT resulted very effective for all BD acute depressive and manic episodes not responding to conventional pharmacologic management.

